# Manufacture of electrical and magnetic graded and anisotropic materials for novel manipulations of microwaves

**DOI:** 10.1098/rsta.2014.0353

**Published:** 2015-08-28

**Authors:** P. S. Grant, F. Castles, Q. Lei, Y. Wang, J. M. Janurudin, D. Isakov, S. Speller, C. Dancer, C. R. M. Grovenor

**Affiliations:** 1 Department of Materials, University of Oxford, Parks Road, Oxford OX1 3PH, UK; 2 International Institute for Nanocomposites Manufacturing, WMG, University of Warwick, Coventry CV4 7AL, UK

**Keywords:** metamaterials, manufacture, processing, transformation optics, spatial transforms

## Abstract

Spatial transformations (ST) provide a design framework to generate a required spatial distribution of electrical and magnetic properties of materials to effect manipulations of electromagnetic waves. To obtain the electromagnetic properties required by these designs, the most common materials approach has involved periodic arrays of metal-containing subwavelength elements. While aspects of ST theory have been confirmed using these structures, they are often disadvantaged by narrowband operation, high losses and difficulties in implementation. An all-dielectric approach involves weaker interactions with applied fields, but may offer more flexibility for practical implementation. This paper investigates manufacturing approaches to produce composite materials that may be conveniently arranged spatially, according to ST-based designs. A key aim is to highlight the limitations and possibilities of various manufacturing approaches, to constrain designs to those that may be achievable. The article focuses on polymer-based nano- and microcomposites in which interactions with microwaves are achieved by loading the polymers with high-permittivity and high-permeability particles, and manufacturing approaches based on spray deposition, extrusion, casting and additive manufacture.

## 1. Introduction

Transformation optics is a theoretical tool for the design of devices that control light and may be used to determine the spatial variation of the relative permittivity *ε* and permeability *μ* required in a structure in order to produce a given, desired effect on the propagation of electromagnetic radiation [1,2]. The early experimental realization [3] of a cloaking device—a one-off fabrication and operating over a narrow band of frequencies—demonstrated the utility of the concept and suggested new opportunities for the development of electromagnetic device technology. However, to enable such opportunities to be realized, materials and manufacturing processes must be further developed, in which regard there lies two key challenges. First, the required *ε* and *μ* determined by the transformation optics procedure can give rise to values that are unachievable in any single material, or that are required to be highly anisotropic. Therefore, a ‘palette’ of bulk materials with a wide range of controllable electromagnetic properties needs to be established. Second, even if bulk materials with the requisite values of *ε* and *μ* are available, the ability to pattern the required variations in these properties as a function of position should be available to produce graded changes in properties, discrete step variations, or anisotropy. Therefore, new material–manufacturing combinations should be developed—preferably inexpensive and scalable—so that spatial variations in electromagnetic properties can be readily patterned to a specified resolution. Currently, the options are limited so that, while theory and simulation continue to advance, practical demonstrations and validation are either somewhat inelegant, non-scalable or simply unavailable. In this paper, we focus on developments at this materials–manufacturing interface relevant to possible future transformation optics applications. The focus is not on classic metamaterials approaches of regular arrays of resonating elements, but instead on manufacturing approaches to ‘all-dielectric’ and magnetic property variations. In each case, the advantages and limitations of the materials–process combinations are described.

## 2. Materials

Polymer-based composites can combine the processability of the polymer matrix with the electromagnetic properties of discrete filler particles according to established mixing rules [4,5]. The criterion for the composites to behave as a homogeneous medium with respect to the propagation of electromagnetic radiation is that the wavelength within the medium be much larger than the scale of the particles or particle agglomerates. With well-dispersed particles of typical dimension 10^−6^ to 10^−4^ m subject to microwaves of free-space wavelength greater than 10^−2^ m, this criterion is easily satisfied, leading to non-resonant bulk materials suitable for broadband applications. In all cases, the effective electromagnetic properties of the composite may be changed by varying the fraction of particles, up to an upper limit usually dictated by the mechanical robustness of the composite. The resulting bulk composites can generally be readily characterized in terms of permittivity and permeability at a range of microwave frequencies using a number of techniques, including stripline, rectangular waveguide, coaxial airline and split post dielectric resonator [6–9] methods.

The different types of polymer-based composites may be categorized into three groups, ‘high-*ε*’ (real permittivity), ‘magnetic’ and ‘superconducting’, with most attention in the literature given to the former and least to the latter. For ‘high-*ε*’ composites that underpin the all-dielectric approach to wave manipulation, the aim is to achieve a conveniently processable dielectric material with a relatively large real part of the relative permittivity (*ε*′). Common bulk polymers, such as polyethylene (PE) and polypropylene (PP), are readily processed but provide only *ε*′=2–3 at approximately 10 GHz [10,11], limiting their use in transformation optics devices. On the other hand, while ceramics such as BaTiO_3_ are brittle and much less versatile with respect to processing, they have bulk permittivities at microwave frequencies that can be orders of magnitude larger than those of polymers [12,13]. Consequently, by dispersing micro-scale particles of BaTiO_3_ in a polymer matrix, processable materials with *ε*′ up to 20 in the frequency range 12–18 GHz can be obtained [14–18].

For magnetic composites, again, the goal is to develop readily processable materials but with a relatively large real part of their relative permeability *μ*′. Common polymers, being non-ferromagnetic, have real permeability *μ*′∼1. Through the addition of ferromagnetic metals such as Fe, and ferrites such as Ni_0.4_Zn_0.6_Fe_2_O_4_, as the filler material, composites with *μ*′ up to approximately 10 at microwave frequencies can be obtained. For both high permittivity and magnetic composites, the large density difference between fillers and matrix can lead to difficulties in maintaining an adequate dispersion across large volumes of composite materials during processing. Sub-micrometre filler particles generally suffer from a tendency to agglomerate, and these small sizes can also have reduced dielectric or magnetic response when compared with bulk behaviour, although the magnitude of these reductions can often be difficult to quantify [19].

**Figure 1.**
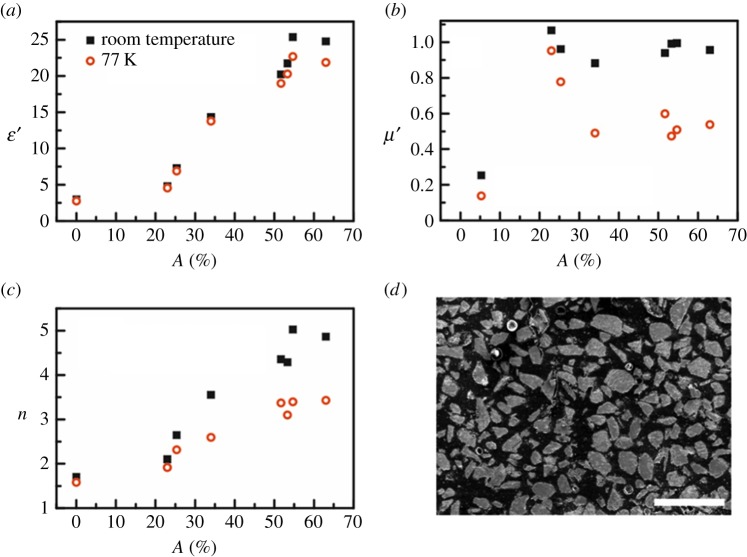
Electromagnetic properties at 15 GHz of a superconducting composite composed of YBCO particles in an epoxy matrix. (*a*) Real part of the relative dielectric permittivity *ε*′ as a function of particle loading by area fraction *A*, at room temperature and in the superconducting state at a temperature of 77 K. (*b*) Real part of the relative permeability *μ*′. (*c*) The quantity 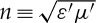. (*d*) Typical cross-sectional electron micrograph of the superconducting composites (scale bar, 500 μm).

Less-studied superconducting composites offer the potential for strongly temperature-dependent electromagnetic properties (as the material is cooled into, or heated out of, the superconducting state) with 0<*μ*′<1 in the superconducting state. For example, the variation of *ε*′ and *μ*′ for a series of cast epoxy-based composites containing different fractions of superconducting yttrium barium copper oxide (YBCO) particles at room temperature and 77 K (in liquid nitrogen) are shown in figure 1. The data at 77 K was obtained by submerging the waveguide measurement jig in liquid nitrogen. In accordance with previous studies [20,21], the quantity 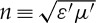 could be controlled by both the YBCO fraction and temperature, as shown in figure 1*c*, where *n* is related to the refractive index, but ignoring the imaginary components *ε*′′ and *μ*′′, which tend to be relatively lossy in these composites; for example, the acrylonitrile butadiene styrene (ABS)—YBCO composite had a loss tangent at 15 GHz of 0.025 at 0.2 vol% YBCO. The increase in *n* with YBCO filler fraction derived from the increase of *ε*′ with filler fraction shown in figure 1*a*, in accordance with the effective medium theory of Bruggeman [5], which provided a good fit to the effective permittivity data for YBCO volume fractions up to 50% using *ε*′=55 for the filler particles (note, considerably smaller than the bulk permittivity of BaTiO_3_ of at least several hundreds). As expected, the effective permittivity did not change significantly on cooling to 77 K. However, figure 1*b* shows there was a reduction in *μ*′ with temperature, in turn causing changes in *n*. The relative permeability at room temperature was 1 at all filler fractions, but the YBCO became diamagnetic below a transition temperature of 93 K owing to the Meissner effect, giving *μ*′<0.5 for the most heavily loaded composites, and the ability to tune *n* between 5 and 3.5 using temperature.

The polymer matrix can be either a thermoset (such as an epoxy resin) or a thermoplastic (PP, ABS, polystyrene (PS)), the final choice of which relates to the required processing route and final device specifications. Homogeneous dispersions of particles can be obtained by a number of methods, depending on the polymer matrix and the processing route: a good dispersion of microparticles in an epoxy is typically obtained via sonication, or mechanical mixing monomers and particles before subsequent polymerization during processing. Particle dispersion in thermoplastics may be readily obtained by mechanical mixing at an elevated temperature in the polymer melt—for example, via screw extrusion. Alternatively, if a thermoplastic can be readily dissolved, the microparticles may be dispersed in solution, with the solvent subsequently evaporated to re-precipitate the polymer around the particles.

**Figure 2.**
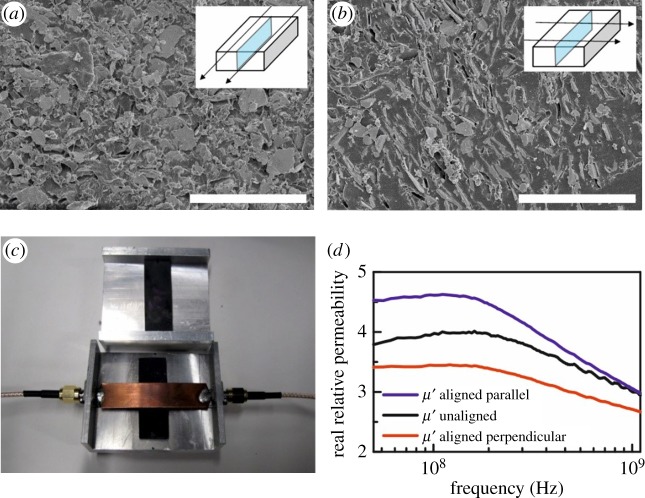
Magnetically aligned anisotropic composites composed of Fe flakes in epoxy. (*a*,*b*) Electron micrographs show preferential alignment of the flakes due to the applied magnetic field. The insets indicate the direction of the magnetic field (black arrows) with respect to the sample geometry and the orientation of the exposed section (pale blue) (scale bars, 50 μm). (*c*) Photograph of the samples and the stripline measurement apparatus used to retrieve the effective permeability of the composite. (*d*) The real part of the relative permeability as a function of frequency for three types of sample, showing the induced anisotropy of the magnetically aligned samples.

Bulk composites are typically isotropic in terms of *ε*′ and *μ*′ but these properties in a composite can be contrived to be anisotropic with suitable processing: for example, the alignment of magnetic particles in an external magnetic field applied to an epoxy-based Fe-containing composite during curing, as shown in figure 2. Anisotropic superstructures may also be contrived using arrangements of bulk isotropic materials: for example, if an isotropic composite is formed into a regular array of uniformly oriented rods, it behaves as a homogeneous, but anisotropic, material for wavelengths much larger than the distance between the rods [22].

## 3. Processing techniques

### (a) Spray deposition

**Figure 3.**
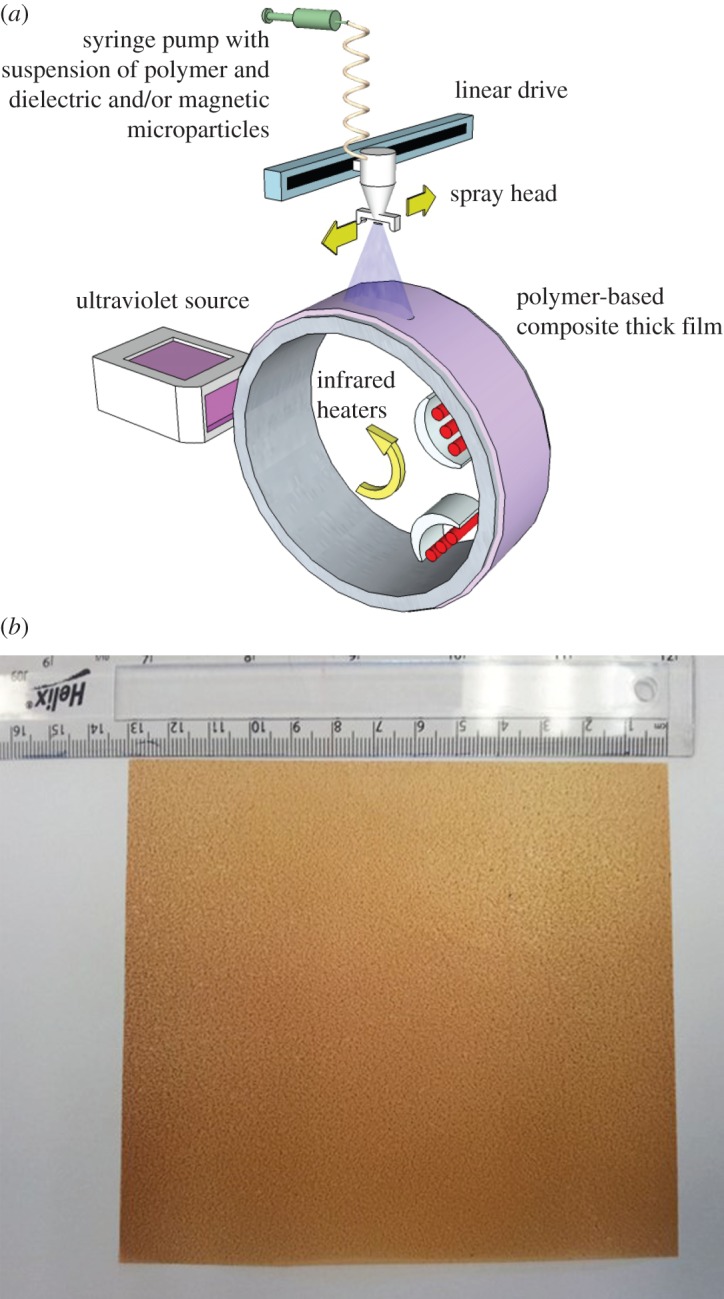
(*a*) Schematic diagram of the spray deposition equipment used to produce dielectric and magnetic particle-containing polymer-based films. (*b*) A 13× 13 cm PFA–Fe_3_O_4_ composite sheet made by spray deposition followed by curing.

Spray deposition offers the potential of a low-cost, scalable manufacturing for the production of polymer-composite films and multilayers containing dielectric, magnetic or combinations of nano- or microparticles. A schematic of the apparatus used to produce composite films is shown in figure 3*a*. In this process, a critical step is the development of a stable suspension of polymer and particles so that constant fractions are deposited over the length of the deposition and uniform properties result. Suspension stability is usually achieved by ultra-sonication (for times of up to several hours), possible small additions of surfactant, and restricting the particle diameter (typically less than 5 μm). To produce a suspension of low enough viscosity for spraying, a fugitive carrier is used that either dissolves the polymer or suspends small polymer particles stably, while also suspending the filler particles. The suspensions are then syringe (up to 10 ml min^−1^) or peristaltic (5–50 ml min^−1^) pumped at a controlled rate into a compressed air atomiser to form a stable spray of 10–200 μm diameter droplets, each of which likely contains filler particles. On deposition, the fugitive liquid phase (often and preferably water, but sometimes organic solvents) evaporates almost immediately as the rotating drum substrate is maintained at elevated temperature using infrared heaters. Depending on the polymer, an ultraviolet (UV) light source can be used to polymerize *in situ* monomer (e.g. acrylate) as it is precipitated from the evaporating solvent, and the suspended particles are engulfed directly into the polymer film [23]. In an alternative approach, 80 nm particles of thermoplastic perfluoroalkoxy (PFA) have been suspended alongside one-dimensional carbon materials, and the resulting film then heated in a belt furnace to cure thermally, rather than using UV light, to form a robust high-permittivity composite film [24]. The same approach can be used for magnetic suspensions and figure 3*b* shows a sprayed PFA-based composite film containing magnetic Fe_3_O_4_ nanoparticles, in this case 13×13 cm in size and approximately 60 μm thick.

A range of expressions—or ‘mixing laws’—are available to predict composite effective permittivity and permeability of films and bulk materials from the individual component properties and their fractions, as described in [23], with the logarithmic Lichtenecker mixing law the most widely applied for composite permittivity *ε*′_c_:

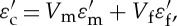
where *V*_m_ and *V*_f_ are the matrix and filler volume fraction, and *ε*′_*m*_ and *ε*′_f_ are the real permittivity of matrix and filler. Comparison with experimental data shows mixing laws such as Lichtenecker or Bruggeman [5] generally approximate well to composite properties for minority filler additions, but significant deviations can occur where there is significant agglomeration [25] or where the filler has a high aspect ratio [24].

**Figure 4.**
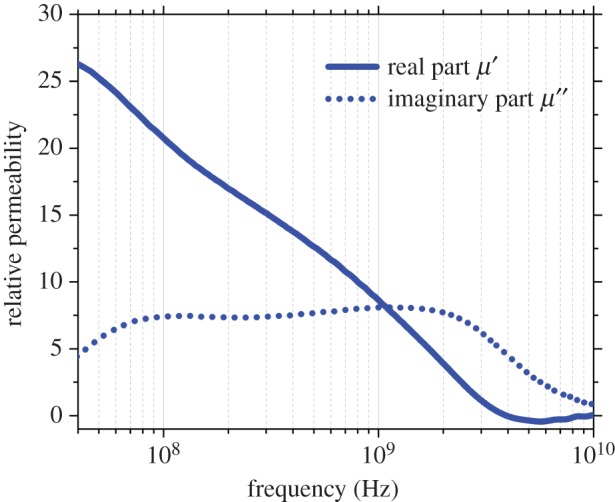
Permeability characterization of a strip of magnetic composite film that has been wound into a ring on a central spool, comprising an elastomeric matrix with embedded Fe flakes, measured by a simple coaxial technique and corrected for residual air gaps.

To obtain measurements of *ε* and especially *μ* for films such as those shown in figure 3*b* over a range of microwave frequencies can be difficult. However, an approach has been developed in which films can be tightly wound on a central spool to form a ring shape, which is then inserted snugly into a coaxial line measurement arrangement [26]. Winding is usually done by hand but results in entrapped air gaps between layers of dielectric that subsequent density measurements, for example by a water displacement method, suggest may occupy up to 20 vol%. Although this coaxial arrangement is simple and allows ready measurement of reflection and transmission behaviour of the film across a broad band of frequencies, it is necessary to apply analysis or a model that accounts for these inevitable air gaps. Figure 4 shows data in the frequency range 30 MHz to 10 GHz from an elastomeric film containing magnetic Fe flakes similar to the film in figure 3*b* but with a stronger magnetic response, and where the measured permeability has been adjusted using an analytical layered capacitor model that considers the arrangement as alternating concentric rings of dielectric composite and air, and accounts for the presence of 12 vol% air gaps [26].

### (b) Extrusion

**Figure 5.**
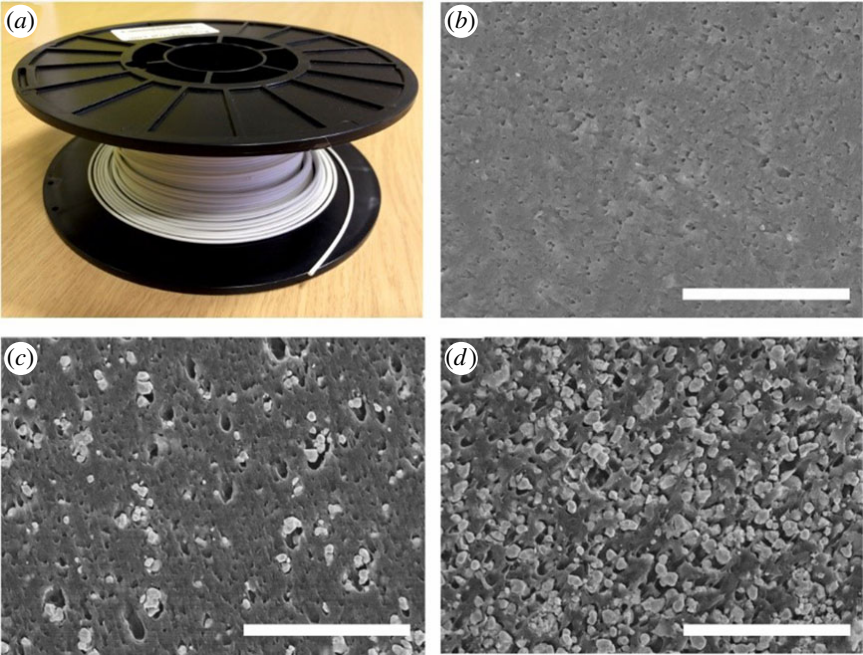
(*a*) A reel of ‘high-*ε*’ filament composed of BaTiO_3_ microparticles in ABS polymer, produced in-house by premixing followed by extrusion. (*b*–*d*) Cross-section scanning electron micrographs of filaments with 0, 30 and 75 wt% BaTiO_3_ microparticles, respectively (scale bars, 10 μm).

Extrusion of polymer composites can produce filament that is then used as the feedstock for additive manufacture [27–30]. Figure 5*a* shows a reel of ‘high-*ε*’ filament of BaTiO_3_ microparticles dispersed in ABS matrix, made in-house for use with a fused deposition modelling (FDM) three-dimensional printer. Figure 5*b*–*d* shows the microstructure of these filaments with particle loadings of 0, 30 and 75 wt% BaTiO_3_. Extrusion can also be used to produce much larger composite sheet or planar geometries of constant thickness, similar in some ways to spray deposition, but at a higher material flow rate. While it would be most convenient if the required fractions of polymer and filler could be loaded directly into a screw or similar extruder to produce composite sheet directly, a premixing stage, for example by mechanical granulation or dissolving the polymer in a solvent and mixing with the powder followed by drying, is generally preferred if excessive agglomeration is to be avoided and/or a consistent filler fraction across significant volumes of material, e.g. tens of metres of sheet or filament, is to be achieved.

**Figure 6.**
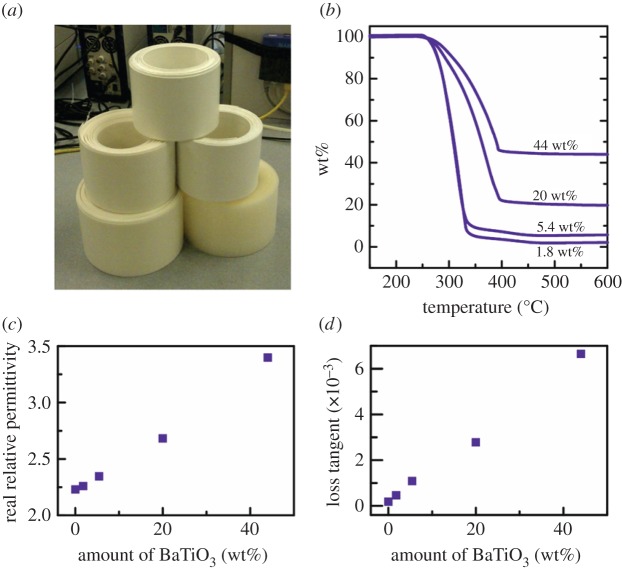
Approximately 7 cm wide extruded sheets of BaTiO_3_ microparticles in PP. (*a*) Coiled extruded composite sheets with various particle loadings. (*b*) The weight percentage BaTiO_3_ in the composite sheets determined by thermogravimetric analysis, showing how the polymer is removed at high temperature as a gas and the residual BaTiO_3_ weight can be estimated. (*c*) Real part of the relative permittivity as a function of particle fraction. (*d*) Dielectric loss tangent as a function of particle loading. Measurements in (*c*,*d*) were taken at ≈15 GHz using a split post dielectric resonator.

Figure 6*a* shows extruded and coiled sheets of PP with various loading fractions of BaTiO_3_, and where the nominal fractions of BaTiO_3_ were then confirmed using thermogravimetric analysis in figure 6*b*. Figure 6*c*,*d* shows there was a modest and systematic increase in effective permittivity at ≈15 GHz, measured using a split post dielectric resonator, together with an increase in loss as the fraction increased. The composite sheets may be subsequently hot-pressed together to form a bulk composite material, or laminates of graded electromagnetic properties in one direction.

### (c) Casting

**Figure 7.**
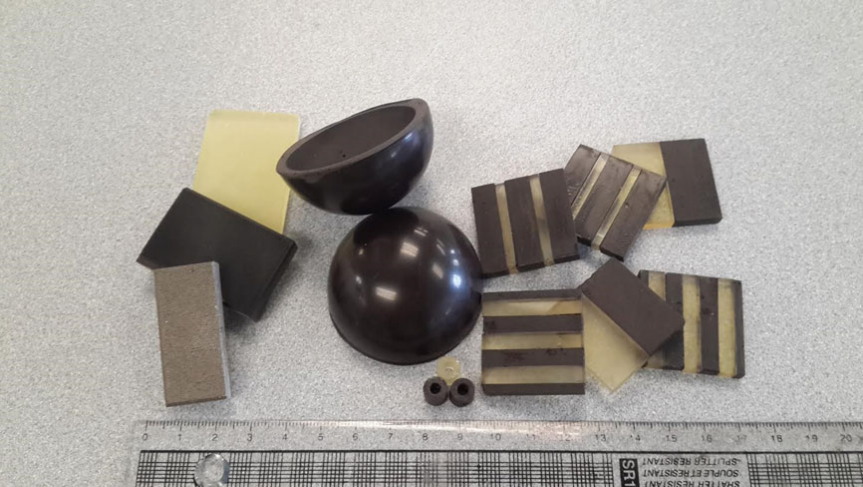
Epoxy-based NiZn ferrite-containing magnetic composite examples showing the flexibility of casting, including: hemispherical shells for potential application in electrically small folded spherical helix antenna, tiles comprising polymer only (transluscent) and heavily filled (black) regions of varying relative thickness for anisotropic magneto-dielectric substrates in low-profile antenna, and blocks and rings suitable for stripline and coaxial line measurements, respectively.

Casting is a straightforward, inexpensive and widespread manufacturing technique, whereby an object is formed by introducing a liquid material into a suitably shaped mould before subsequent solidification or polymerization. Because of its convenience, casting has been used routinely in the preparation of bulk composites for electromagnetic applications. Microparticle fillers are typically dispersed in epoxide monomers and the resulting mixture poured into a silicone mould before polymerization and resulting solidification at an elevated temperature (approx. 60°C) overnight. If needed, even at up to high loading fractions of 60 vol%, the cast samples can be conventionally machined (with care) to a high tolerance for a wide range of designed applications, as shown in figure 7. Casting has been used to produce impedance-matched materials with relatively high and equal permittivity and permeability that are promising for applications in telecommunications [31]; more than one type of filler material may also be introduced in this approach to produce hybrid composites for superior electromagnetic performance, e.g. extending the frequency at which a useful magnetic response (permeability more than 1) can be achieved [32].

**Figure 8.**
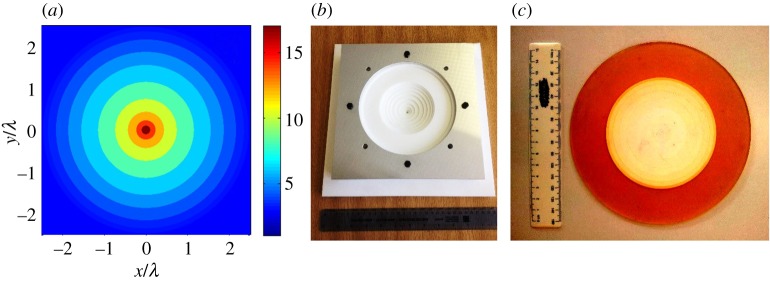
Fabrication of graded dielectric structures via casting of epoxy-based composites with controlled fractions of high-permittivity BaTiO_3_ particles. (*a*) A continuous but un-manufacturable radial profile of relative permittivity as a function of position from transformation optics is approximated to a manufacturable discretized profile. (*b*) A series of moulds are used to produce cast concentric rings with different filler fractions chosen to provide the desired radial profile of permittivity. (*c*) A graded device is fabricated, from all-epoxy at the outer ring through to BaTiO_3_/epoxy composite inner rings with *ε*′ up to 17.

Whereas spray deposition and extrusion produce only planar geometries, casting in principle can produce complex three-dimensional shapes with homogeneous properties. Sequential casting operations have also been developed to produce step-graded all-dielectric structures based on transformation optics designs, as shown in figure 8. In this case, a continuous decrease in permittivity of a flat ring was desired to act as a surface wave beam-splitter, from a permittivity of 17 (achieved using a BaTiO_3_ fraction of 40 vol% in epoxy) in the centre of the circular device reducing to 2.5 (epoxy only) at a radius of 80 mm. For manufacturability, the continuous radial variation from design simulations was approximated to a discrete step variation as shown in figure 8*a*, which was then achieved by sequentially casting concentric rings of epoxy-based composites loaded with progressively lower BaTiO_3_ fractions towards the outermost ring, starting with the central core (figure 8*b*,*c*) and then into concentric moulds of increasing radius.

### (d) Additive manufacture

Additive manufacturing (AM) has long been used as a rapid prototyping technique but is increasingly being explored for the direct manufacture of low-rate, high-value components. The technique has been used for the fabrication of electromagnetic devices that operate at microwave frequencies [33–37]. Typical of the structures studied by AM are three-dimensional inter-penetrating designs of the deposited polymer and air (designed-in voids), in order to achieve effective grading or anisotropy in properties. Most of these studies have used common (usually polymeric) materials, not specifically chosen for their inherent permittivity or permeability properties but because of their compatibility with the AM process, but may suffer from high loss tangents. For example, polymers that are used for FDM—one of the most popular and affordable processes—such as ABS are limited to relative permittivities of approximately 3 at 10 GHz [38], a dielectric loss tangent of up to 0.01 and relative permeabilities of essentially 1.

Further progress in the AM of more capable electromagnetic devices based on transformation optics can be expected to be facilitated by the development of AM-compatible materials but with more contrived or designed electromagnetic properties. For example, the concentric ring design made by sequential casting in figure 8 might be achieved much faster by AM and in a single operation—if printable materials of sufficient dielectric contrast of 2.5 to approximately 17, and offering sufficiently low loss, were available and reliable. For example, figure 6*d* suggested that loss tangents can be restricted to approximately 5×10^−3^ at 40 wt% BaTiO_3_ if a PP matrix were to be adopted for AM. Moreover, AM becomes particularly enabling where such gradient properties are achieved in three dimensions.

So far, AM structures and devices have been based very largely only on spatial variations in permittivity, and otherwise the permeability is near unity. However, where permeability might also be varied spatially and simultaneously, more design freedom is enabled so that, for example, reflection losses can be minimized by providing impedance *Z*=*Z*_0_(*μ*/*ε*)^1/2^, where *Z*_0_ is the permittivity of free space, alongside spatial variations in permittivity.

Using bespoke composite filaments produced in-house, as shown previously in figure 5, the FDM approach has been used to produce ‘high-*ε*’, ‘magnetic’ and ‘superconducting’ composites directly. In the FDM process, composite filament is delivered by a motorized drive system at a calibrated flow rate into a heated nozzle where it is first melted and then extruded through a small orifice and thus directly onto the top surface of the forming component. The component sits on a build platform and the relative motion between component and print head is controlled by a pre-programmed pattern. At the end of each finished layer of deposition, the build platform is lowered and the next layer then printed. Re-optimization of the FDM parameters, e.g. nozzle temperature, feed rate and traverse speed of the head, is required for reliable printing when using composite filament.

**Figure 9.**
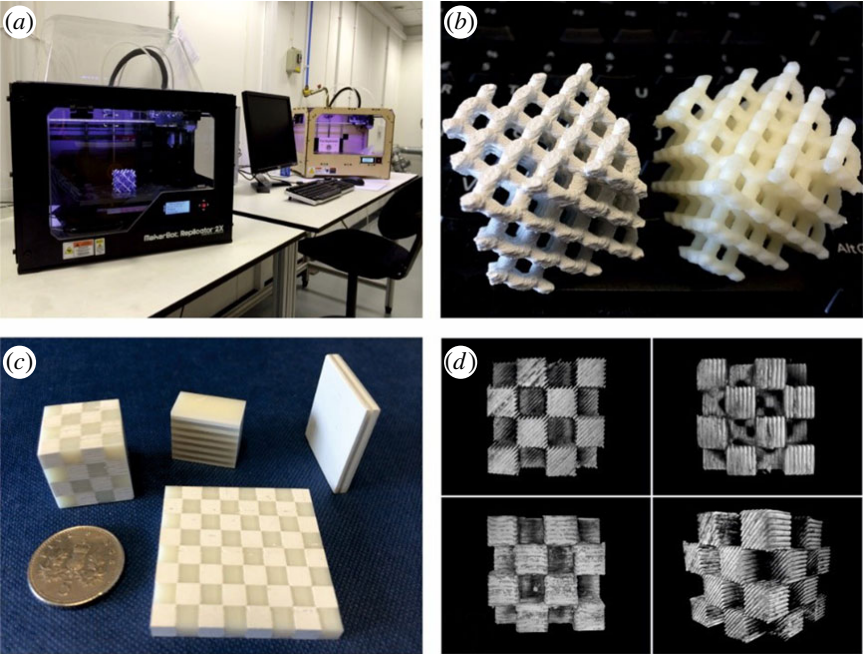
Additive manufacture of ‘high-*ε*’ composites composed of BaTiO_3_ microparticles in ABS. (*a*) The three-dimensional printers used for manufacture. (*b*) A diamond-like lattice composed of polymer only with low permittivity (right) and a polymer+BaTiO_3_ composite with high permittivity (left). (*c*) Samples composed of alternating areas of polymer and polymer+BaTiO_3_ printed using dual extrusion nozzles. (*d*) X-ray computed tomography images of the ‘chequerboard’ cube in (*c*), revealing the internal layered structure.

Figure 9 shows (*a*) low-cost three-dimensional printers adapted for the printing of composite filaments, (*b*) two diamond-like lattice structures printed from ABS (right) and ABS+60 wt% BaTiO_3_ (left) and (*c*) ‘chequerboard’ and layered slabs and cubes comprising well-controlled volumes of ABS-only and ABS–BaTiO_3_ composite. These structures are similar to those assembled by hand from epoxy and machined ceramic rods to produce resonance effects at microwave frequencies [39] but where AM approaches could be used to adjust much more readily the spacing of layers and the volume fraction of filler in the layers. In figure 9*c*, a loaded and a non-loaded polymer filament was used alternately and layer thicknesses were approximately 0.2 to 1 mm. By inter-layering of unfilled and filled filaments at the finest resolution of approximately 100 μm, the relative number of each layer can be used to contrive the effective local volume fraction so that a range of effective permittivities at the meso-scale might be realized, lying in the range between that of the filled and the unfilled filament.

In order to assess the presence of air gaps between printed layers—a typical problem for AM that undermines target permittivity and permeability—X-ray tomography was applied to the cube in figure 9*c* and the results in a series of orientations are shown in figure 9*d*. Contrast between the array of sub-cubes is produced by the more strongly X-ray-absorbing BaTiO_3_-loaded regions. The presence of the BaTiO_3_ allows the discrete layers of the build process to be resolved and suggests there may be limited fine-scale fusing, i.e. critical wetting, interfacial mixing and adherence between subsequent vertical layers, under these particular conditions.

**Figure 10.**
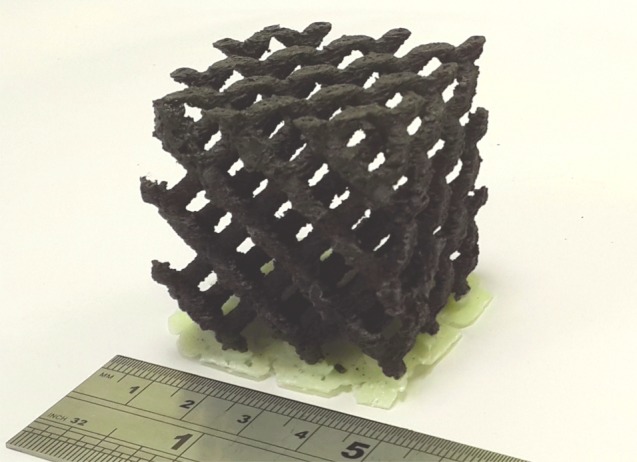
Lattice structure produced by FDM additive manufacture using ABS polymer with 10 vol% NiZn ferrite filament.

Figure 10 shows the same diamond-like lattice structure as in figure 9*b* but now realized by additive manufacture in an ABS+10 vol% NiZn ferrite composite, which provided a real permeability of approximately 2 up to 100 MHz for 25 vol% ferrite, and of approximately 1.3 up to 1 GHz for 10 vol% ferrite when printed in bulk.

The limitations of printed composites relate to the maximum fraction of filler that can be used before the filament becomes too brittle and fragile to handle. For ‘high-*ε*’ composites based on BaTiO_3_, this upper limit is typically 75 wt% BaTiO_3_, which can provide a real permittivity of approximately 10 in the frequency range 12–18 GHz. Comparison with the upper limit achievable by casting of approximately 17, also with approximately 75 wt% BaTiO_3_ but dispersed in epoxy in the central core of figure 8*c*, suggested the presence of any inter-layer voids in AM will undermine achievable composite properties. However, limited use (to avoid inducing further loss) of surfactants and plasticizers [30,40,41] may be able to increase printable volume fractions in AM, and the development of higher-intrinsic-permittivity but low-loss polymers (such as PP shown in figure 6*d* for extrusion) suitable for AM, along with process parameters that ensure optimized inter-layer fusing, will all have a significant effect on enhancing the achievable effective properties.

## 4. Summary and future outlook

Transformation optics provides designs for novel and useful manipulations of microwaves but can also demand unrealistic absolute values of permittivity and permeability, and their spatial variation. Where the most extreme properties have been developed using resonant phenomena, narrowband and high-loss performance is usually problematic. In the microwave domain, spatial variations in EM properties are typically in the millimetre to the many centimetre range, making a range of both traditional and new fabrication processes relevant. Polymers offer excellent processability to produce filaments, thin films, sheets, bulk materials and three-dimensional shapes, but to be useful for transformation optics, various types of fillers must be added, and the range of resulting materials is rapidly expanding. Additive manufacture offers significant potential for the all-dielectric approach to transformation optics devices, but both bespoke materials developments and robust manufacturing technology are at an early stage. Where multi-materials can be combined at fine scale but over large-scale component dimensions by AM, new functionalities to enable transformation optics designs are very likely. However, AM capabilities must be improved to eliminate air gaps, to manage distortions and surface finish to provide geometric conformance, and to automatically detect and correct print defects.
